# Schistosomiasis vaccine discovery using immunomics

**DOI:** 10.1186/1756-3305-3-4

**Published:** 2010-01-28

**Authors:** Patrick Driguez, Denise L Doolan, Alex Loukas, Philip L Felgner, Donald P McManus

**Affiliations:** 1 Molecular Parasitology Laboratory, The Queensland Institute of Medical Institute, Herston, Queensland 4006, Australia; 2 School of Population Health, University of Queensland, Herston, Queensland 4006, Australia; 3 Molecular Vaccinology Laboratory, The Queensland Institute of Medical Institute, Herston, Queensland 4006, Australia; 4 Helminth Biology Laboratory, The Queensland Institute of Medical Institute, Herston, Queensland 4006, Australia; 5 Department of Medicine, University of California, Irvine, CA 92067, USA

## Abstract

The recent publication of the *Schistosoma japonicum *and *S. mansoni *genomes has expanded greatly the opportunities for post-genomic schistosomiasis vaccine research. Immunomics protein microarrays provide an excellent application of this new schistosome sequence information, having been utilised successfully for vaccine antigen discovery with a range of bacterial and viral pathogens, and malaria.

Accordingly, we have designed and manufactured a *Schistosoma *immunomics protein microarray as a vaccine discovery tool. The microarray protein selection combined previously published data and in silico screening of available sequences for potential immunogens based on protein location, homology to known protective antigens, and high specificity to schistosome species. Following cloning, selected sequences were expressed cell-free and contact-printed onto nitrocellulose microarrays. The reactivity of microarray proteins with antisera from schistosomiasis-exposed/resistant animals or human patients can be measured with labelled secondary antibodies and a laser microarray scanner; highly reactive proteins can be further assessed as putative vaccines. This highly innovative technology has the potential to transform vaccine research for schistosomiasis and other parasitic diseases of humans and animals.

## Review

Schistosomiasis causes significant morbidity and mortality in the developing world with recent studies indicating that the geographic extent and burden of the disease exceeds official estimates [1]. Praziquantel-based chemotherapy has achieved some success in controlling the disease but is not an optimal strategy due to its inadequate impact on reducing long-term transmission [1]. Despite the mass chemotherapy programs, schistosome reinfection rates and prevalence continue to be unacceptably high, with rebound prevalence and morbidity an inevitable consequence if ongoing interventions are not sustained [2,3]. Along with other options, long-term protection afforded by vaccination will be necessary for the future control and possible elimination of schistosomiasis. The currently available vaccine antigens were discovered empirically using attenuated schistosome larvae, protective monoclonal antibodies, or by analysis of human antibody and cytokine responses to recombinantly-derived proteins [4]. These identified vaccine molecules may, however, lack the required efficacy because: 1) the vaccine-induced protective immunity generated in animal models may not translate to humans; 2) there is uncertainty about the type of human response most appropriate for protective immunity; and 3) the antigens may not be expressed on the schistosome apical surface, and will not therefore be exposed to the host immune system [2,4].

Key to the identification of new target vaccine molecules and high throughput antigen discovery are the recently published complete genomes of *Schistosoma japonicum *and *S. mansoni *[5,6], and related post-genomic research on the schistosome proteome, transcriptome, glycome and immunome [7,8]. The amalgamation of the information provided by these data sets, together with consideration of the host-parasite immune response in the field of immunomics, promises to result in more rapid and promising antigen discovery and the development of an effective vaccine for schistosomiasis [9-11].

Conventional proteomic studies on schistosomes identified proteins from male and female worms, different life-cycle stages, and parasite fractions and excretions that were separated by one or two dimensional (1/2D) gel electrophoresis (GE) and/or liquid chromatography followed by mass spectrometry (MS). Exposed proteins on the schistosome surface can be further characterised using biotinylated reagents, infection sera and/or by enzymatic stripping [12]. For example, 71 sero-reactive adult *S. haematobium *worm antigens were identified using 2D GE of soluble parasite fractions, labelling with resistant human sera, and identification by MS [13].

However, due to the limitations of MS detection and protein extract preparation and separation, often only the most abundant cytosolic and possibly least immunologically meaningful proteins can be identified by this procedure [7,12]. An immunomics protein microarray provides a convenient method that avoids some of the limitations inherent in other proteomic approaches but allows profiling of the host immune response to parasite antigens in a high throughput manner.

Since the first application of immunomics for vaccine discovery, antigens for ten microbial pathogens have now entered clinical or preclinical development [9]. Immunomics-based approaches typically combine in silico genome screening followed by high throughput protein expression, and purification and immunological testing of selected proteins [9]. The genome mining techniques include reverse vaccinology and epitope mapping, i.e. the prediction of potential virulence factors or secreted/surface proteins, and immunogenic T- or B-cell epitopes [9,10,14]. A further modification to the immunomics selection process is the incorporation of comparative or pan-genomics, and structural-genomics [9,11].

**Figure 1:**
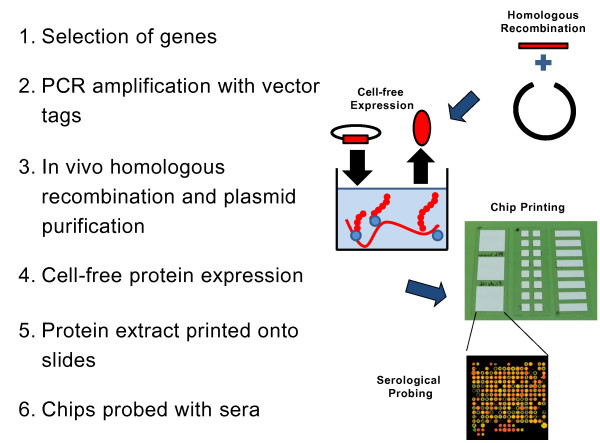
**Design, construction and probing of the *Schistosoma *immunomics protein microarray**.

An immunomics approach has not hitherto been utilised in schistosome (or any other metazoan parasite) antigen discovery. Consequently, with collaborators at the University of California, Irvine (UCI), we have used a previously published strategy [15] to design and construct a *Schistosoma *protein microarray for the identification of novel anti-schistosome vaccine candidates. The design, manufacture and probing of the microarray is illustrated in Figure 1.

**Figure 2:**
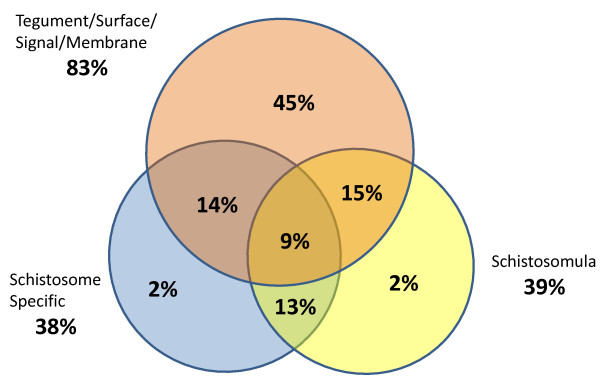
**Composition of the proof-of-concept schistosome protein microarray**. Red circle, tegumental/surface/signal peptide/membrane proteins from various stages; blue circle, proteins specific to *Schistosoma *species; and yellow circle, proteins specific to schistosomula.

As proof-of-concept for the approach, a subset of potentially immunogenic open reading frames (ORFs) were selected for expression and printing from publically available coding sequences for *S. japonicum *(214 selected) and *S. mansoni *(63 selected). These sequences were chosen from bioinformatic, proteomic and transcriptomic data using the following criteria: high sequence homology among the two schistosome species; expression in the immunologically vulnerable schistosomulum larval stage; predicted or known to be localised on the parasite surface; and limited sequence similarity with mammalian homologues (Figure 2). In addition, *S. japonicum *homologues of *S. mansoni *vaccine candidates and surface proteins (30 ORFs) were also selected as were a large proportion of hypothetical and proteomically uncharacterised proteins (77 ORFs). Primers were designed with 20 base pairs specific to each transcript and 20 base pairs specific to the expression vector (5'-ACGACAAGCATATGCTCGAG; 3' - ATTAACCTTATGAAAATATT). Sequences were PCR-amplified from schistosome cDNA templates and incorporated into the pXi T7 bacterial vector by homologous recombination. Of the sequences, 88% (244) were able to be PCR- amplified and these resultant plasmids were purified, and the inserts verified by PCR and by sequencing.

**Figure 3:**
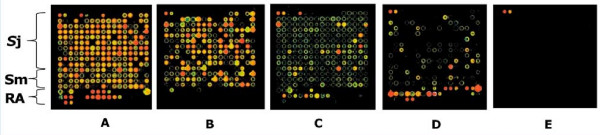
**Probing of the schistosome protein microarray**. The protein microarray includes cell-free *S. japonicum *(Sj) and *S. mansoni *(Sm) proteins as well as known recombinant antigens (RA) that were expressed conventionally *in vivo *in bacteria and yeast. The protein microarray was probed with monoclonal antibodies against the N-terminal poly-histidine tag (A) and C-terminal hemagglutinin tag (B); with pooled sera from Swiss outbred mice after a 7 week infection with *S. japonicum *(C); *S. mansoni *(D); and naïve mouse serum (E).

A total of 222 (80%) high-yielding plasmids with correct inserts were expressed in an in vitro cell-free *Escherichia coli *system (Roche RTS 100), and the protein extracts contact-printed without purification onto nitrocellulose glass FAST^® ^slides. In addition, known *S. japonicum *and *S. mansoni *recombinant antigens (serial dilutions of 17 constructs including tetraspanins, annexins, Sm29, Sj29, and expression tags alone) expressed in *E. coli *or *Pichia pastoris *were printed in each microarray. Controls on the microarray included nine no-DNA negative controls -in vitro expression solution without plasmid template, three PBS only controls, and positive controls (four serial dilutions of: pooled human, mouse, guinea pig, goat, cat and chicken IgG antibodies, Jackson ImmunoResearch; and the EBNA1 protein of Epstein-Barr virus). The printed in vitro expressed proteins were quality checked using antibodies against incorporated N-terminal poly-histidine (His) and C-terminal hemaglutinin (HA) tags (Figure 3A &3B), and showed high protein expression efficiency. The printed protein microarray is amenable to probing with anti-sera, pre-treated with *E. coli *lysate, to prevent non-specific binding of immunoglobulins to bacterial proteins on the microarray. Serological reactivity is determined using fluorophore-labelled secondary antibodies and detected using a laser array scanner. The signal intensities are transformed and normalised using the vsn statistical package http://www.r-project.org and the background signal determined from the average of the no-DNA controls within each microarray. Positive signal intensity is defined as two standard deviations above the background signal [16]. Preliminary probing of the schistosome immunomics protein microarray with pooled sera from Swiss mice infected for 7 weeks with *S. japonicum *(Figure 3C), *S. mansoni *(Figure 3D), and naïve mouse sera (Figure 3E) is shown, demonstrating specific recognition of schistosome antigens by the murine host. The recognised antigens are a combination of in vitro-expressed hypothetical, surface and structural proteins as well as recombinant antigens expressed in bacteria and yeast. It is noteworthy that our preliminary analysis indicates some antigens are commonly reactive with sera from mice infected with either *S. japonicum *or *S. mansoni*. This approach can be readily extended for the identification of putative anti-schistosome vaccine targets using resistant versus susceptible infection sera from animal models and/or humans from endemic areas, subsequent to preclinical vaccine efficacy studies.

This protein microarray technology platform has been now applied to over 30 pathogens including viruses, bacteria and protists (e.g. [17]). The approach allows efficient expression of putative antigens without many of the problems of conventional protein expression and purification. This is exemplified by *Plasmodium falciparum *where high throughput protein expression using bacteria, yeast, insect cells as well as the wheat germ cell-free system results in very low expression efficiency because of its rare codon usage and high A + T content. However, using a similar platform to the *Schistosoma *protein microarray described here, >93% expression efficiency was achieved for a similar proof-of-concept study of 250 P. falciparum proteins with 14 new potential vaccine targets being identified [18].

Compared with conventional proteomics approaches, an immunomics protein microarray can efficiently express many more potential antigens that are selected using known or predicted surface location and are readily identified after serological screening. A comparison of the characteristics of proteomics approaches with immunomics protein microarrays is presented in Table 1. Immunomics provides a powerful addition to antigen discovery, but the technology does have some drawbacks summarised in Appendix 1. For example, proteins expressed by the *E. coli *cell-free system may lose some post-translational modifications and tertiary protein structure [18]. Further, different microarray antigens cannot be compared with each other and protein levels within each microarray feature are difficult to quantify [16].

**Table 1:** A comparison of the characteristics of immunomics protein microarrays with conventional proteomic approaches

Characteristic	Protein Microarrays	Conventional Proteomic Approaches
**Protein Structure**	Can be limited	Present, depending on methods

**Protein Abundance**	Not affected	Low abundance proteins lost

**Protein Identification**	Not affected	Difficult

**Pathogen Protein Extraction**	Not applicable (uses DNA/cDNA)	Difficult

**Protein localisation**	Not affected	Membrane proteins difficult to extract and detect

**Technical difficulty**	Low	High

Nevertheless, this antigen discovery approach will be readily adaptable to other parasites as more genome sequence information and post-genomic data become available. We anticipate that such microarray platforms will facilitate the identification of novel vaccine candidates that may not have been revealed using conventional methodologies, thereby providing a valuable approach for vaccine discovery.

## Competing interests

PF has patent applications related to this technology and an equity interest in Antigen Discovery Inc. The other authors declare that they have no competing interests

## Authors' contributions

PD, PF and DPM conceived the idea and planned the experiments. PD executed the experiments and analysed the data. PD, PLF, DLD, AL and DPM wrote the manuscript.

## Appendix 1

Advantages and disadvantages of immunomics protein microarray technology.

### Advantages

Proteome-wide, selective expression of full-length proteins including insoluble and membrane proteins

Protein expression can be determined from C- and N-terminal tags

High throughput screening of antibody responses from experimentally immunized or naturally exposed individuals and animal models

Small serum sample volume (1 microliter) required for immunoscreening

### Disadvantages

Some loss of protein tertiary structure and post-translational modifications

Reactivity is not directly comparable between microarray antigens

Microarray protein levels cannot be quantified

Antibodies against *E. coli *proteins in cell-free extract must be blocked
